# HTLV-1 molecular epidemiology in central Australia: Two distinctive HTLV-1 Subtype C lineages in Indigenous Australians

**DOI:** 10.1186/1742-4690-11-S1-P56

**Published:** 2014-01-07

**Authors:** Olivier Cassar, Lloyd Einsiedel, Philippe V Afonso, Antoine Gessain

**Affiliations:** 1 Institut Pasteur, Unité d’Epidémiologie et Physiopathologie des Virus Oncogènes, Département de Virologie, Paris, France; 2 CNRS, UMR 3569, Paris, France; 3 Flinders University/Northern Territory Rural Clinical School, Alice Springs Hospital, Northern Territory, Australia

## 

HTLV-1 infects approximately 5-10 million people worldwide. It is widely distributed, with clusters of high endemicity in certain geographic areas or ethnic groups. Seven main HTLV-1 subtypes have been described and the Melanesian/Australian subtype C is only found in Oceania. In Australia, HTLV-1 is endemic with ATLL, TSP/HAM, bronchiectasis, ID and severe scabies cases reported. However, HTLV-1 clinical significance is debated and only one partial HTLV-1 sequence from an Aboriginal Australian (MSHR-1) is available. Therefore, establishing a large HTLV-1 sequence database is essential for the understanding of the epidemiology and pathogenesis of this virus in Indigenous people. Samples were obtained from 23 HTLV-1 patients originated from four broad geographical and language-based areas. Sequence comparison indicated that the 23 new HTLV-1 LTR and Tax-Gag sequences belong to the subtype C as MSHR-1 strain. Phylogenetic analyses evidenced two main clades (Solomon Islands/Vanuatu *versus* Australia) within subtype C. Interestingly, the Australian clade can be itself divided into two clusters: the first comprising strains characterized among most of the Indigenous Australians from the North and the second including a majority of individuals originating from the South and Central Australia. Thus, there is a clear evidence of a specific HTLV-1 clustering according to the childhood residence of the HTLV-1 infected individuals, and subsequently their language group affinity. Taking into account that non-human primates have never been found in Australia, we believe that these HTLV-1 subtype C variants have been present among the first ancestors of modern Indigenous Australians for several tens of millennia.

